# Detection of circulating melanoma cells in choroidal melanocytic lesions

**DOI:** 10.1186/s13104-015-1420-5

**Published:** 2015-09-17

**Authors:** Manuel F. Bande, Maria Santiago, Laura Muinelo-Romay, Maria Jose Blanco, Purificacion Mera, Carmela Capeans, Maria Pardo, Antonio Piñeiro

**Affiliations:** Ocular Oncology Unit, Servizo de Oftalmoloxía, Complexo Hospitalario Universitario de Santiago, Universidade de Santiago de Compostela, Santiago de Compostela, Spain; Liquid Biopsy Analysis Unit, Translational Medical Oncology Group, Health Research Institute of Santiago (IDIS), Complexo Hospitalario Universitario de Santiago de Compostela, Santiago de Compostela, Spain; Grupo Obesidómica, Instituto de Investigación Sanitaria de Santiago (IDIS), Hospital Clínico Universitario de Santiago (CHUS/SERGAS), Santiago de Compostela, Spain

## Abstract

**Purpose:**

To detect and quantify circulating tumour cells (CTCs) in peripheral blood of patients with uveal melanoma primary non-metastatic tumours, and to analyze the possible relationship between CTCs and clinical risk factors.

**Methods:**

Prospective study with two clinical groups: 4 patients diagnosed with choroidal nevus and 8 patients with choroidal melanoma prior to treatment. A single sample of 7.5 mL of peripheral blood was taken and the CTCs were isolated using a CellSearch system that captures positive cells for the CD146 antigen (MUC18).

**Results:**

None of the patients with choroidal nevus showed CTCs in peripheral blood. More than one CTC/7.5 mL was detected in 50 % of patients with choroidal melanoma prior to treatment. The higher level of CTC cells in peripheral blood (3/7.5 mL) was detected in the patient with the larger choroidal melanoma which also presented extrascleral extension and epithelioid pathology.

**Conclusion:**

Performing an analysis with the CellSearch system allows to quantify the choroidal melanoma CTCs in peripheral blood. This finding highlights the potential usefulness of this technique to achieve the correct stratification and monitoring of the treatment.

## Background

Despite the successful treatment of uveal melanoma (UM) primary tumors, patients remain at risk of developing metastases for more than 20 years after the initial diagnosis. In the Collaborative Ocular Melanoma Study (COMS), Kaplan–Meier analyses estimated that the 2-, 5-, and 10-year metastasis rates were 10, 25, and 34 %, respectively. However, only 0.24 % of patients exhibited detectable metastases at the time of diagnosis [1].

The CellSearch system (Veridex) was developed to identify and quantify CTCs in the peripheral blood by immunomagnetic isolation and inmunohistochemical detection. This platform obtained the Food and Drug Administration (FDA) clearance for the CTC enumeration in patients with breast, colon, or prostate cancers [2, 3]. Despite that CellSearch system has been previously used in the detection of CTC in patients with metastatic uveal melanoma [4]; this technology has not been tested for non-metastatic/localized UM and choroidal nevi until now.

Our group investigated the possibility of detecting and quantifying CTCs using the semiautomatic CellSearch system in the peripheral blood of patients CTCs in the patients with primary/localized UM. Further, in this preliminary study we examined the relationship between the presence of CTCs, clinical parameters and disease-free survival.

## Methods

**Fig. 1:**
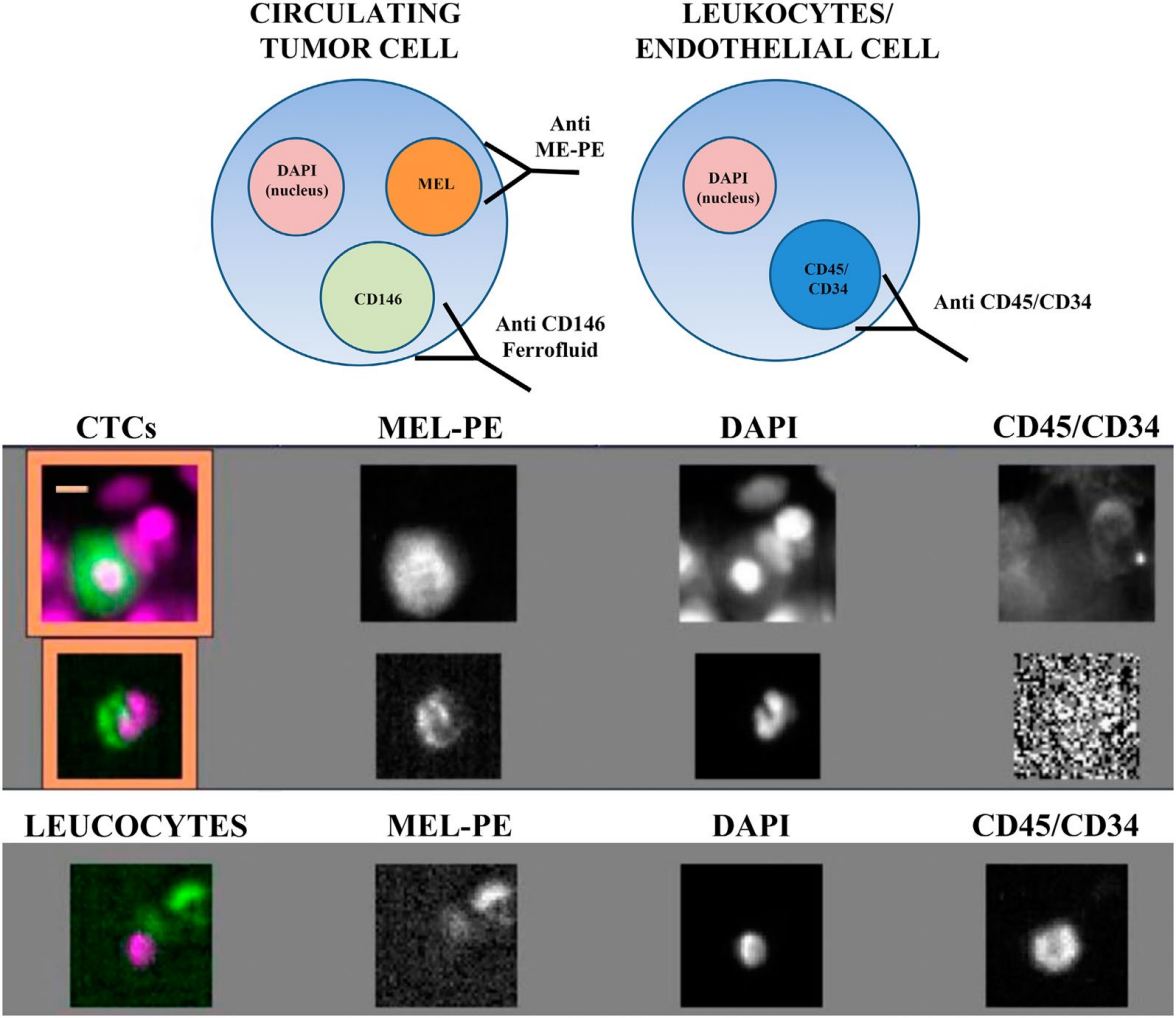
Immunophenotype of melanoma circulating tumor cells (DAPI+, MEL+, CD45−/CD34−) and a leukocyte/endothelial cell (DAPI+, MEL−, CD45+/CD34+). The *images* beneath the cells indicate how the CTCs appear by fluorescence microscopy. The *orange scale bar* is 5 μm in length

12 Patients (8 UM and 4 choridal nevus) diagnosed at the Ocular Oncology Unit (Sevicio de Oftalmología, Santiago de Compostela, Spain) were included in the study after informed consent according to the Declaration of Helsinky. This study was also approved by the Comité Ético de Investigación Clínica de Galicia. Peripheral blood (7.5 mL, CellSave preservative tube, Veridex) was extracted at identical venipuncture points from each patient. Melanoma cells were detected by the CellSearch system (Veridex, USA) as previously described [5, 6]. Briefly, melanoma cells were isolated with magnetic beads coated with anti-CD146 antibody. Then the CD146-expressing cells were stained with the fluorescent nucleic acid dye 4′,6-diamidino-2-phenylindole dihydrocloride (DAPI) and with a combination of fluorescent antibodies against high-molecular-weight melanoma-associated antigen (MEL), CD34 and CD45 to distinguish melanoma cells from leukocytes and endothelial cells (Fig. 1). Cell were considered CTCs when they have oval morphology and were positive for DAPI, MEL and negative for CD34 and CD45. Absence of metastatic melanoma at the time of blood sampling was confirmed by clinical evaluation, routine biochemistry and liver ultrasonography in all patients. The comparisons were done by using the Mann–Whitney U test. The correlations were done by Pearson’s correlation analysis.

## Results

Among the eight patients with non-treated choroidal melanoma, 50 % displayed more than one CTC per 7.5 mL of blood. No significant correlation was shown between the CTC positivity and clinicopathological parameters, including the diameter of the largest tumor basal (LBD), the height of the tumor. The average patient follow-up was 25 months (min 16; max 27 months). The most important descriptive observation that emerged from this preliminary study was obtained from the largest choroidal melanoma, which demonstrated extrascleral extension (patient 1, Table 1). This patient presented the greatest number of CTCs and he was the only patient who presented metastatic liver disease at 12 months follow up. Moreover, four choroidal nevi (<2-mm height and <8-mm base) were tested, in which none CTCs were found.

**Table 1:** Description of clinical features

Num	Thickness (mm)	Base (mm)	Location	Escleral extension	CTCs
1	11.46	10.67	Superior	+	3
2	10.16	11.40	Nasal	−	0
3	9.10	16.00	Nasal	−	0
4	6.11	13.70	Temporal	−	0
5	4.81	10.37	Temporal	−	2
6	4.12	10.56	Optic nerve	−	1
7	3.31	8.47	Nasal	−	2
8	2.46	12.14	Temporal	−	0

## Discussion

Circulating tumor cells (CTCs) are associated with the development of metastatic disease. In recent years, there has been considerable interest in the detection of disseminated tumor cells in the peripheral blood and bone marrow of patients with solid tumors. This interest is due in part to the identification and quantification of CTCs by improved methods, such as immunomagnetic/immunohistochemistry [7–9] and reverse-transcriptase polymerase chain reaction (RT-PCR) [10]. Various studies have included PCR to quantify UM-associated mRNAs, such as tyrosinase and the melanoma antigen recognized by T-1 cells (MART-1/MLANA) [11]. These studies have not provided additional information beyond that of standard clinical approaches [12, 13]. This deficiency appears to be due to a lack of technique standardization, possible sample contamination, and the inability to directly quantify tumor cells.

On the other hand, we have not found a clear relationship between CTCs and tumor size or histological type in the studied patients. We also found that CTCs were not detected in the peripheral blood of 50 % of patients with UM. This last point may be due to a better prognosis for these patients, but it may also owe to low detection sensitivity. The enrichment of the CellSearch System procedure with UM specific antibodies, in combination with anti-ME-PE, would significantly enhance the assay sensitivity.

This study has shown that the CellSearch system is appropriate to detect CTCs in patients with primary/localized UM. The ability to quantify and characterize CTCs in patients with UM is already a clinical reality with the immunomagnetic method. This system is semiautomatic and can be used in a clinical laboratory, making it reproducible in a standardized manner. Moreover, this methodology would allow to perform further molecular characterization by probing with FITC conjugated antibodies for expression of other markers, and also the cells could be analyzed by FISH and PCR for gene expression and mutation analysis [14].

The correlation among the number of CTCs and the clinical factors such as tumor size and scleral invasion described in one of the UM patients in the present study is promising and suggest future applications of this technology. Additionally, this fact is supported by a very recent investigation describing the presence of CTCs in metastatic UM [4].

## Conclusions

This finding demonstrates potential utility of CellSearch System for correct stratification, prognostic estimation, establishment of early therapies, and evaluation of response to different treatments in patients with UM.

## Abbreviations

UM: uveal melanoma
CTCs: circulating tumor cells
RT-PCR: reverse-transcriptase polymerase chain reaction
MART-1/MLANA: tyrosinase and the melanoma antigen recognized 143 by T-1 cells
LBD: largest tumor basal
MEL: melanoma-associated 144 antigen
DAPI: acid dye 4′,6-diamidino-2-phenylindole dihydrocloride
FDA: Food and Drug Administration
